# Integrative Network Analysis Unveils Convergent Molecular Pathways in Parkinson's Disease and Diabetes

**DOI:** 10.1371/journal.pone.0083940

**Published:** 2013-12-20

**Authors:** Jose A. Santiago, Judith A. Potashkin

**Affiliations:** The Cellular and Molecular Pharmacology Department, The Chicago Medical School, Rosalind Franklin University of Medicine and Science, North Chicago, Illinois, United States of America; University of Hawaii Manoa, United States of America

## Abstract

**Background:**

Shared dysregulated pathways may contribute to Parkinson's disease and type 2 diabetes, chronic diseases that afflict millions of people worldwide. Despite the evidence provided by epidemiological and gene profiling studies, the molecular and functional networks implicated in both diseases, have not been fully explored. In this study, we used an integrated network approach to investigate the extent to which Parkinson's disease and type 2 diabetes are linked at the molecular level.

**Methods and Findings:**

Using a random walk algorithm within the human functional linkage network we identified a molecular cluster of 478 neighboring genes closely associated with confirmed Parkinson's disease and type 2 diabetes genes. Biological and functional analysis identified the protein serine-threonine kinase activity, MAPK cascade, activation of the immune response, and insulin receptor and lipid signaling as convergent pathways. Integration of results from microarrays studies identified a blood signature comprising seven genes whose expression is dysregulated in Parkinson's disease and type 2 diabetes. Among this group of genes, is the amyloid precursor protein (*APP*), previously associated with neurodegeneration and insulin regulation. Quantification of RNA from whole blood of 192 samples from two independent clinical trials, the Harvard Biomarker Study (HBS) and the Prognostic Biomarker Study (PROBE), revealed that expression of *APP* is significantly upregulated in Parkinson's disease patients compared to healthy controls. Assessment of biomarker performance revealed that expression of *APP* could distinguish Parkinson's disease from healthy individuals with a diagnostic accuracy of 80% in both cohorts of patients.

**Conclusions:**

These results provide the first evidence that Parkinson's disease and diabetes are strongly linked at the molecular level and that shared molecular networks provide an additional source for identifying highly sensitive biomarkers. Further, these results suggest for the first time that increased expression of *APP* in blood may modulate the neurodegenerative phenotype in type 2 diabetes patients.

## Introduction

Parkinson's disease and type 2 diabetes are among the most prevalent diseases affecting the aging population. Recent findings have revealed convergent molecular and biological pathways that link both diseases. Mitochondrial dysfunction, endoplasmic reticulum stress, inflammation and alterations in glucose metabolism are disrupted in both diseases [1]. Exposure to environmental factors and genetic susceptibility are thought to be involved in the etiology of both diseases. Accordingly, most cases of Parkinson's disease and type 2 diabetes are considered sporadic with 5–10% attributed to known genetic factors. Several shared genetic connections between diabetes and Parkinson's disease have recently been identified. For example, regulation of expression of *PINK1*, previously associated with Parkinson's disease [2], is altered in skeletal muscle of type 2 diabetes patients [3]. Likewise, DJ-1, an antioxidant protein with reduced expression in Parkinson's disease is also reduced in pancreatic islets of type 2 diabetes patients and increases during aging under non-diabetic conditions [4]. To date, there is no modifying agent or preventive treatment available but commonly prescribed drugs to treat diabetics have shown promise in Parkinson's disease clinical trials [5], [6]. Neuroprotection conferred by these drugs is attributed to the targeting of the inflammatory pathways. In addition to inflammation, impaired insulin signaling and glucose metabolism, hallmarks of diabetes, may play a role in the development and progression of Parkinson's disease, therefore understanding the molecular framework that links both diseases is expected to facilitate the development of novel therapeutic strategies.

High-throughput methods have successfully identified thousands of genetic associations with Parkinson's disease and type 2 diabetes. However, the large amount of data is difficult to integrate and it is often problematic to interpret the underlying functional disease mechanism based on the annotation of a single gene. Complex diseases such as Parkinson's disease and type 2 diabetes are affected by many genes that may act synergistically to contribute to disease development perhaps by participating in common biological pathways. Network biology has emerged as a powerful tool for the interpretation and integration of genomic data to understand disease-disease and gene-disease associations [7]–[11]. In this context, integrated network-based approaches have been used to identify pathways and susceptibility genes associated with Parkinson's disease and type 2 diabetes. For example, using an integrative systems biology approach, axon guidance, focal adhesion, and calcium signaling were identified among the most significant pathways in Parkinson's disease [12]. Likewise, using a network approach a set of genes associated with insulin signaling and nuclear receptors were identified in type 2 diabetes models [13]. In addition, analysis of metabolite-protein networks identified biomarkers for pre-diabetes [14].

Here we employ an integrated network approach to dissect the molecular networks and dysregulated pathways shared between Parkinson's disease and type 2 diabetes. Our network approach utilizes a random walk based algorithm (RWR) to quantitatively prioritize genes according to their topological distance and functional relatedness with known disease genes in the functional linkage network (FLN) [15]. The use of the FLN as a platform to rank potential disease-related genes is based on the premise that a group of genes known to contribute to a particular disease phenotype are usually functionally related. The weight of each link between a pair of genes represents the likelihood that the linked genes share common biological processes. In addition, we integrate data from previous microarray studies to identify a whole blood signature characteristic of Parkinson's disease and type 2 diabetes. In order to translate these results into a clinically relevant tool for disease diagnosis, we evaluate the expression of *APP* in blood of Parkinson's disease patients in samples from two independent clinical trials. In this study we provide evidence that Parkinson's disease and type 2 diabetes are highly interconnected at the molecular level. Further, this study supports the idea that complex diseases like Parkinson's disease and type 2 diabetes may result as a consequence of perturbations in shared molecular networks.

## Methods

Genes associated with Parkinson's disease and type 2 diabetes were retrieved from the GWAS catalog (http://www.genome.gov/gwastudies/). Genes with a genome-wide significance level of p<10^−08^ were included in this study. A random walk algorithm with restart (RWR) was performed using Gene Prioritization and Evidence Collection (GPEC), a Cytoscape 2.8.3 plugin [16]. We used the weighted and undirected human FLN for this analysis [17]. Confirmed genes associated with Parkinson's disease and type 2 diabetes obtained from the GWAS catalog, were specified as the training set (Tables S1 and S2). The candidate set included neighboring genes within a topological distance of less or equal than 1 in the FLN. The RWR algorithm is formally defined elsewhere [15]. Briefly, the RWR moves from a seed node to a randomly immediate neighboring node or returns to the start node with a probability α at each step [15]. To perform the RWR, we set the restart probability α to 0.5 and candidate genes were scored and ranked. RWR scores for prioritized genes are listed in Table 1 and Table S3. Biological and functional analysis was performed using the Genemania plugin [18].

**Table 1:** RWR scores for the top 20 ranked genes.

Rank	Gene	Score
1	CD63	4.45E-03
2	CDK1	4.26E-03
3	USHBP1	2.18E-03
4	RAF1	1.43E-03
5	PKN1	1.21E-03
6	MAPK1	9.54E-04
7	RHOA	8.98E-04
8	CREBBP	8.71E-04
9	COPB1	7.83E-04
10	AKT1	7.68E-04
11	ARF1	7.68E-04
12	BRAF	7.59E-04
13	RALGDS	7.01E-04
14	ARF3	6.99E-04
15	APP	6.97E-04
16	POU4F1	6.90E-04
17	ROCK2	6.76E-04
18	MAPK3	6.75E-04
19	PRKCA	6.54E-04
20	ROCK1	6.48E-04

### Ethics statement and PROBE and HBS study participants information

The Institutional Review Boards of Rosalind Franklin University of Medicine and Science approved the study protocol. Written informed consent was received from all participants. 96 individuals including 50 Parkinson's disease patients (mean Hoehn and Yahr scale 2, Table 2) and 46 healthy age-matched controls were enrolled in the HBS. Details of patient and controls recruitment, clinical assessments, and biobanking in the HBS study population have been reported in part elsewhere [19] and http://www.neurodiscovery.harvard.edu/research/biomarkers.html. As an independent replication set, we used 51 Parkinson's disease patients (mean Hoehn and Yahr scale of 2) and 45 healthy age-matched controls enrolled in the PROBE Study (#NCT00653783). Clinical diagnosis of Parkinson's disease was based on the United Kingdom Parkinson's Disease Society Brain Bank criteria [20]. Healthy controls had no history of neurological disease and a Mini-Mental State Examination (MMSE) test score higher than 27. Details of patient and controls recruitment, clinical assessment, inclusion and exclusion criteria have been reported in part elsewhere [21]. Clinical description of study participants is listed in Table 2.

**Table 2:** Clinical characteristics of HBS and PROBE study participants.

HBS			
Disease status	PD	HC	p-value
Number	50	46	>0.5
Age at enrollment (Mean ± SD)	63.12±8.96	64.28±10.42	>0.5
Age of onset (Mean ± SD)	58.75±10.17	N/A	>0.5
Male	31	26	>0.5
Female	19	20	>0.5
BMI (Mean ± SD)	N (16); 22.81±1.54	N (19); 22.26±2.09	>0.5
	OW (22); 27.08±1.35	OW (12); 26.92±1.42	0.0001
	OB (12); 35.65±3.43	OB (15); 33.14±2.98	>0.5
	OW+ OB (34); 30.36±4.82	OW + OB (27); 29.77±3.86	0.01
Hypertension	18	15	>0.5
Diabetes	5	5	>0.5
Hoehn & Yahr (Mean ± SD)	1.97±0.62	N/A	>0.5
**PROBE**			
Number	51	45	>0.5
Age at enrollment (Mean ± SD)	63.16±6.42	65.12±8.60	>0.5
Male	29	24	>0.5
Female	22	21	>0.5
Diabetes	0	1	>0.5
Hoehn & Yahr (Mean ± SD)	2±0.28	N/A	>0.5

BMI is body mass index, N is normal, OW is overweight and OB is obese. BMI was defined by standard measures as normal (N) = 18.5–24.9, overweight (OW) = 25–29.9 and obese (OB) =  30 or greater.

### RNA isolation and real time polymerase chain reactions

Blood was collected and prepared as described using the PAXgene Blood RNA system (Qiagen,Valencia, CA) [22]. Samples with RNA integrity values >7.0 and a ratio of absorbances at 260/280 nm between 1.7 and 2.4 were used in the current study. Primer Express software (Applied Biosystems, Foster City, CA) was used to design the primers. Primer sequences used in qPCR assays are as follows: app; forward: 5′- TTTTCTAGAGCCTCAGCGTCCTA-3′; reverse: 5′- CCCTGGGCTTCGTGAACA-3′, gapdh; forward: 5′-CAACGGATTTGGTCGTATTGG-3′; reverse: 5′-TGATGGCAACAATATCCACTTTACC-3′. The High Capacity RNA transcription kit (Applied Biosystems, Foster City, CA) was used to reverse transcribe 1 µg of total RNA according to the manufacturer's protocol. The DNA engine Opticon 2 Analyzer (Bio-Rad Life Sciences, Hercules, CA) was used for the qPCR reactions. Each 25 µl reaction contained Power SYBR and primers at a concentration of 5 µM. The amplification conditions used are as follows: denature at 95°C for 15 sec, annealing at 57°C for 1 min, extension at 75°C for 45 sec for 45 cycles of amplification. Following the PCR reaction a melting curve analysis was run to confirm that a single product was amplified. PCR products were also run on 1.5% agarose gels to verify specificity. Gapdh was used as a reference gene. Samples were loaded in triplicate. No cDNA template, PD and HC positive controls were run in every experiment. Amplification efficiencies were higher than 90% for each primer set. Expression data was analyzed using the ΔΔCt method.

### Statistical analysis

All analyses were performed with Prism4.0 (Graphpad, La Jolla, CA) and Statistica 8.0 (StatSoft, Tulsa, OK, USA). A student t-test (two-tailed) followed by a Tukey-Kramer post-hoc analysis was used to estimate the significance between PD cases and controls. Linear regression was performed on the expression data adjusting for covariates including, sex and age and BMI in the HBS cohort. Correlation analysis was used to determine if individual variables correlate with each other. Microarray data was analyzed using a Benjamini and Hochberg analysis with a FDR = 0.05. Receiver operating characteristic (ROC) curve analysis was performed to evaluate the diagnostic accuracy of the biomarker. A p-value less than 0.05 was considered statistically significant.

## Results

### Shared molecular network in Parkinson's disease and type 2 diabetes

**Figure 1:**
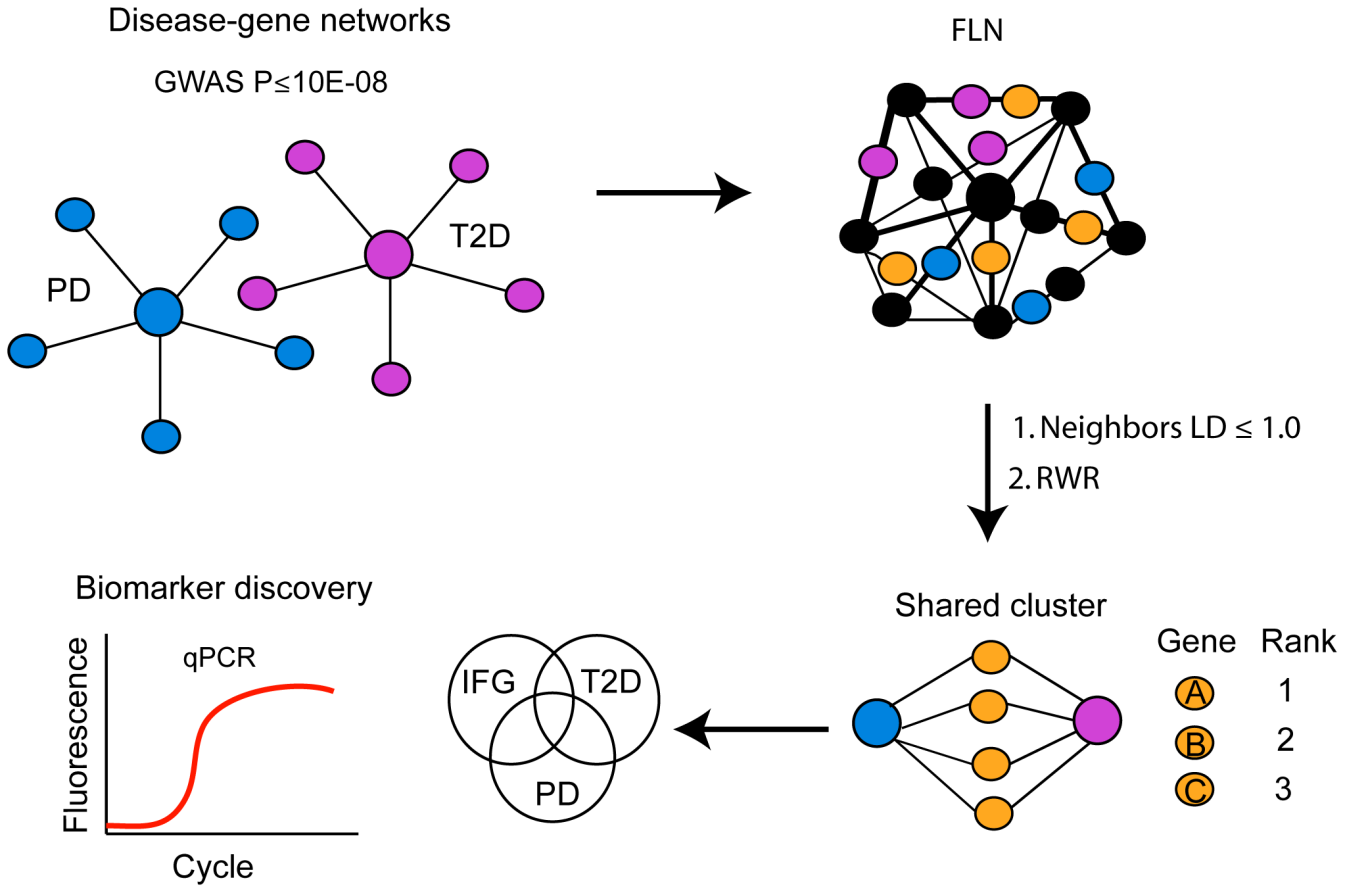
Integrative network approach. Genes with a genome-wide significance of P<10^−08^ or less associated with Parkinson's disease (displayed in blue) and type 2 diabetes (displayed in purple) were included in this study and specified as training genes. A random walk algorithm within the functional linkage network (displayed in gray) was performed to identify candidates genes with a linkage distance (LD) to the training genes of less than or equal to 1 within the FLN. Candidate genes (displayed in orange) were ranked and scored according to their closeness with training genes. Data from microarrays studies in blood of Parkinson's disease, pre-diabetes and Type 2 diabetes patients was analyzed to identify genes dysregulated in both diseases. Quantitative PCR assays were used to validate a potential biomarker in blood of Parkinson's disease patients. Networks were visualized using Cytoscape 2.8.3. PD  =  Parkinson's disease, IFG  =  pre-diabetes and T2D  =  type 2 diabetes.

**Figure 2:**
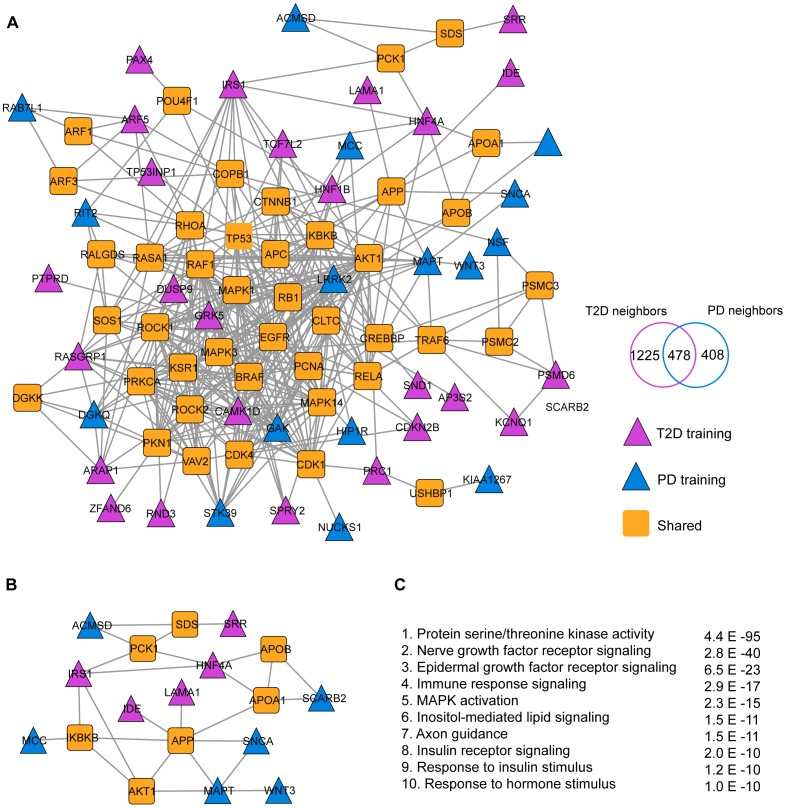
Functional linkage network for Parkinson's disease and type 2 diabetes. **A**. Network visualization of the top 200 shared genes (orange rectangles) closely associated with training genes associated with Parkinson's disease training genes (blue triangles) and type 2 diabetes (purple triangles) within the FLN (displayed in gray). Venn diagram analysis of shared neighboring genes in Parkinson's disease and type 2 diabetes. **B**. Subnetwork visualization of interactions among confirmed Parkinson's disease and type 2 diabetes genes with *APP*. **C**. Overrepresented pathways identified in Parkinson's disease and type 2 diabetes, as retrieved by Genemania. PD  =  Parkinson's disease, IFG  =  pre-diabetes and T2D  =  type 2 diabetes.

In order to investigate the extent to which Parkinson's disease and type 2 diabetes are linked at the molecular level, we performed a RWR algorithm within the human FLN to identify genes associated with both diseases (Figure 1). Genetic associations that confer a risk to Parkinson's disease and type 2 diabetes were retrieved from the GWAS catalog. Only genes with a GWAS significance level of P<10^−8^ were included in this study. A total of 23 genetic loci associated with Parkinson's disease risk were identified in the FLN and specified as training genes (Figure 2A, Table S1). Our test set consisted of neighboring genes with topological distance to the training genes of less than or equal to 1 (LD ≤1). A total of 886 genes were functionally linked to confirmed Parkinson's disease genes. In parallel, using 43 genes associated to type 2 diabetes as training genes, we identified a set of 1,705 neighboring genes (Figure 2A, Table S2). Venn diagram analysis revealed that Parkinson's disease and type 2 diabetes shared 478 neighbors within the FLN (Figure 2A). The top 20 genes prioritized by the RWR are listed in Table 1 and the top 200 ranked genes are listed in Table S3.

Biological and functional analysis of the shared cluster of genes identified pathways associated to the protein serine-threonine kinase activity (p<10^−95^), nerve growth factor receptor signaling (p<10^−40^), immune response signaling (p<10^−17^), MAPK cascade (p<10^−15^), lipid signaling (p<10^−11^), response to insulin stimulus (p<10^−10^), and insulin receptor signaling (p<10^−10^).

Inspection of network topology revealed interesting genetic interactions among well-characterized genes associated with Parkinson's disease and type 2 diabetes. As shown in Figure 2A, multiple type 2 diabetes genetic risk loci are interrelated with Parkinson's disease susceptibility genes throughout the FLN. For example, *APP* interacts with susceptibility genes to type 2 diabetes (*LAMA1* and *IDE*) and genes associated with Parkinson's disease risk including *SNCA* and *MAPT* (Figure 2B).

### A blood signature of Parkinson's disease and type 2 diabetes

**Figure 3:**
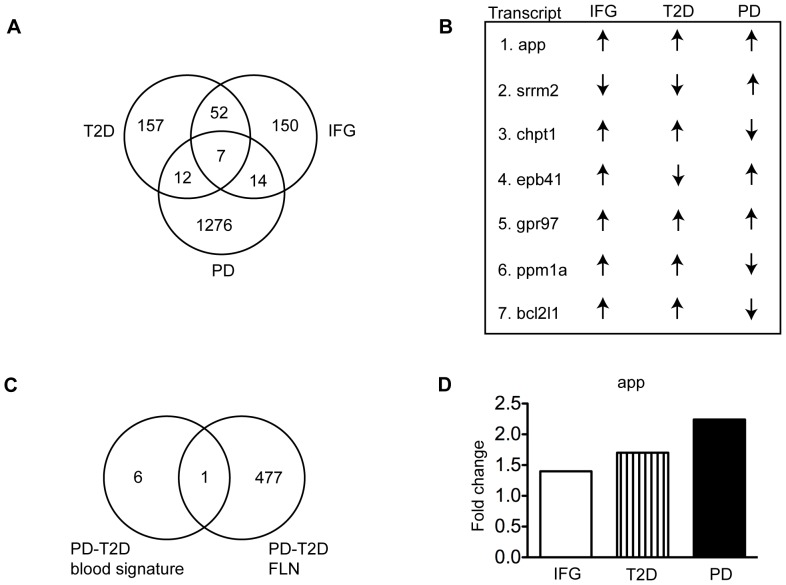
Identification of a blood signature of Parkinson's disease and type 2 diabetes. **A**. Venn diagram analysis of gene expression data from pre-diabetes, type 2 diabetes and Parkinson's disease patients revealed seven genes common to all groups. **B**. Fold change direction for the seven genes signature identified in the microarrays studies. **C**. Venn diagram analysis of the seven genes dysregulated in blood of Parkinson's disease and type 2 diabetes compared to the Parkinson's disease-type 2 diabetes network. **D**. Fold change in mRNA expression of *APP* in microarray studies. PD  =  Parkinson's disease, IFG  =  pre-diabetes and T2D  =  type 2 diabetes.

Impaired insulin signaling and glucose intolerance, hallmarks of diabetes, are implicated in Parkinson's disease [1], [23]. From a system biology perspective, altered expression of genes in peripheral blood may reflect systemic changes observed in both diseases thus providing a better platform to identify disease-specific biomarkers. We interrogated multiple gene expression data sets from independent microarrays studies that used RNA prepared from peripheral whole blood of patients with type 2 diabetes and Parkinson's disease. First, we re-analyzed the study GSE26168 in which changes in mRNA were measured in blood of healthy, impaired fasting glucose, commonly known as pre-diabetes and type 2 diabetes patients. Pair-wise comparisons were performed for each group using a Benjamini and Hochberg analysis with a false discovery rate (FDR) of 0.05 to correct for the occurrence of false positives [24]. In parallel, we re-analyzed microarray data from two previously published studies that compared RNA from whole blood of Parkinson's disease patients compared to healthy individuals (GEO accession numbers: GSE34287, GSE6613). Integration of these microarray studies identified a blood signature of seven transcripts including app, bcl2l1, chpt1, gpr97, ppm1a, and srrm2, common to pre-diabetes, type 2 diabetes, and Parkinson's disease (Figure 3A and B). Only app and gpr97 are upregulated in all groups (Figure 3B). The list of significant genes, fold changes and p-values are listed in Table S4.

We next sought to investigate whether any of the 7 mRNAs were functionally linked to confirmed Parkinson's disease genes in the FLN. Venn diagram analysis identified app as common in both groups (Figure 3C). Interestingly, app mRNA expression was upregulated in pre-diabetes (fold change 1.47, p<0.05)[25] and in Parkinson's disease (2.24, p<0.05)[21] (Figure 3D).

### Biomarker discovery and validation

**Figure 4:**
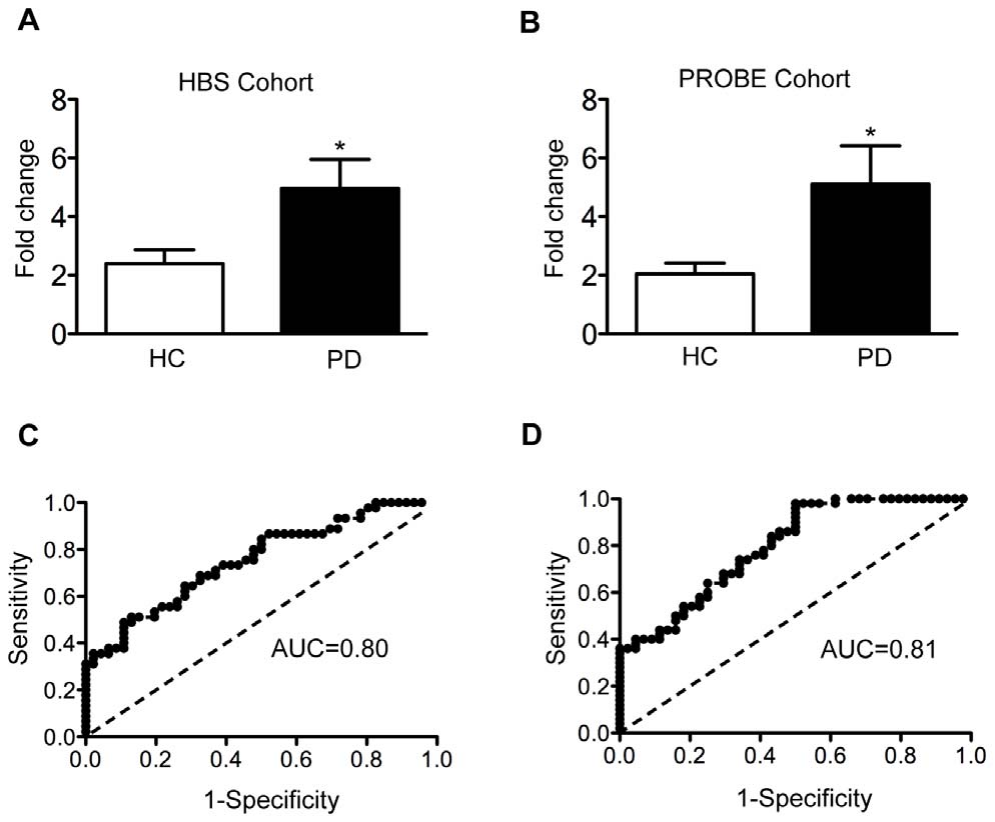
Evaluation of *APP* as a biomarker for Parkinson's disease. **A**. Quantification of app mRNA in blood of Parkinson's disease patients compared to healthy controls in samples from the HBS cohort. **B**. Replication of app mRNA expression in an independent set of samples from the PROBE study. Fold change was calculated using gapdh as a reference gene and healthy controls as a calibrator. Error bars represent standard error. **C**. ROC curve to evaluate the performance of app as a diagnostic biomarker in the HBS cohort. **D**. ROC curve to evaluate the performance of *APP* expression as a diagnostic biomarker and in the PROBE cohort. (*p<0.01). PD  =  Parkinson's disease, HC  =  healthy controls and AUC  =  area under the curve.

Given the numerous molecular links between Parkinson's disease and type 2 diabetes identified in the FLN and microarray studies, we sought to translate these results into a more relevant tool with clinical applicability. Taking into consideration the results generated by integrated network analysis, we evaluated *APP* as a potential biomarker for Parkinson's disease. Relative mRNA levels of *APP* were measured in whole blood of Parkinson's disease patients compared to healthy individuals from samples obtained from two independent clinical trials, the Harvard Biomarker Study (HBS) and the Prognostic Biomarker Study (PROBE). Description of the study participants is listed in Table 2. Gene expression analysis by qPCR revealed that *APP* is significantly upregulated in blood of Parkinson's disease patients compared to healthy controls in the HBS cohort (Mean ± SEM; 4.96±0.98, p = 0.01) and PROBE study (Mean ± SEM; 5.11±1.0, p = 0.01) (Figure 4 A and B). Correlation analysis demonstrated that expression of *APP* was independent of other covariates including age (R = 0.01, p>0.05), sex (R = 0.01, p>0.05), Hoehn and Yahr scale (R = 0.09, p>0.05) in both cohorts of patients and BMI (R = 0.01, p>0.05) in the HBS cohort. Correlation of biomarker expression with medication was not determined since most of the patients with Parkinson's disease were medicated with several drugs and the number of untreated patients was too small to reliably detect a significant change. Receiver operating characteristic (ROC) analysis revealed that app could distinguish Parkinson's disease patients from healthy controls with a diagnostic accuracy of 80% in the HBS cohort (95% confidence interval, 0.65–0.85, AUC = 0.80, p<0.0001) and PROBE study (95% confidence interval, 0.71–0.88, AUC = 0.81, p<0.0001).

## Discussion

The ultimate goal of network biology is to integrate genomic and biological data to aid in the understanding of complex diseases. Ideally, integrative network analysis should enable the discovery of reliable biomarkers and ultimately, therapeutic targets for validation. Here we used an integrative network biology approach to better understand the shared molecular networks in Parkinson's disease and type 2 diabetes. The implementation of the RWR within the FLN to prioritize genes allows us to explore the interconnection between both chronic diseases by considering functional associations. Importantly, the RWR algorithm provides a better performance compared to other network-based algorithms such as the direct neighborhood, graph summarization, Markov clustering and network flow [16], [26].

Integration of genetic networks revealed a molecular cluster comprising 478 genes closely associated with confirmed Parkinson's disease and type 2 diabetes genes. These findings suggest that genes associated with type 2 diabetes can be used to identify genes associated with Parkinson's disease and vice versa. Biological and functional analysis identified the protein serine-threonine kinase activity, nerve growth factor receptor signaling, activation of the immune response, MAPK cascade, lipid signaling, insulin receptor signaling and response to insulin stimulus, as convergent pathways.

Impaired insulin signaling, glucose intolerance and diabetes have been associated with the development and worsening of motor symptoms in Parkinson's disease [27]. Altered expression of genes and metabolites in blood are expected to reflect a systemic response to the impairment of these processes and thereby providing sensitive indicators of disease pathology. In support of this idea, peripheral blood microRNAs are predictive and reflective of metabolic health and disease in type 2 diabetes [25]. Likewise, transcriptional profiling studies from whole blood have identified several molecular signatures associated with Parkinson's disease [21], [22], [28], [29].

Based on these findings, we interrogated several microrray studies from pre-diabetes, type 2 diabetes and Parkinson's disease patients to investigate whether similar changes in gene expression in whole blood exist between both diseases. Integration of these studies revealed a panel of seven genes significantly dysregulated in blood of patients with pre-diabetes, type 2 diabetes, and Parkinson's disease. Among this group, is the serine/arginine repetitive matrix 2 (*SRRM2*), a splicing factor with altered expression in blood and the substantia nigra of Parkinson's disease patients [29]. In the context of aberrant splicing, a subset of splice variants have been associated with Parkinson's disease in samples from two independent clinical trials, thus suggesting a key role of alternative splicing in Parkinson's disease [21], [30].

Another gene with altered expression in blood of pre-diabetes, type 2 diabetes and Parkinson's disease patients is *APP*. Interestingly, the expression of app mRNA in blood is significantly upregulated in pre-diabetes [25] and Parkinson's disease patients [21]. These results suggest that elevated levels of *APP* in blood of type 2 diabetes may be an indicator of neurodegeneration. Therefore, expression of *APP* in blood may be useful to identify type 2 diabetes patients at risk to develop Parkinson's disease.

In order to confirm these findings, we evaluated *APP* expression in blood of patients with Parkinson's disease from two independent cohorts of study participants. Consistent with the microarray data, gene expression levels of *APP* were upregulated in blood of Parkinson's disease patients compared to healthy individuals. Dysregulation of *APP* in blood of Parkinson's disease patients is interesting given its involvement in several neurological disorders. For example, mutations in *APP* linked to familial Alzheimer's disease increase the extracellular concentration of amyloid β protein (Aβ) *in vivo* [31]. More recently, cerebrospinal fluid (CSF) concentrations of Aβ peptides have been widely used to study Alzheimer's disease pathology *in vivo* and their utility to diagnose Parkinson's disease with dementia is under evaluation [32]. In addition to Alzheimer's disease, other neurological disorders including Down's syndrome, autism, and epilepsy are characterized by elevated expression of *APP* [33].

The mechanism by which *APP* increases susceptibility to Parkinson's disease in patients remains unknown. One study found that Aβ peptides enhanced the aggregation of α-synuclein and exacerbated neuronal and motor deficits in a transgenic mouse model [34]. Accordingly, expression levels of Aβ peptides in CSF are associated with motor deficits in early stage Parkinson's disease [35]. Thus, altered processing of Aβ peptides may contribute to neurodegeneration in PD. In a network-based study similar to this, *APP* was identified as a negative regulator of insulin abundance in plasma of mice and a potential link between Alzheimer's disease and type 2 diabetes was suggested [36]. This finding is interesting in light of the recent studies that suggest the involvement of insulin resistance and diabetes in Parkinson's disease [1], [23], [37]. A potential link between APP processing, insulin regulation and neurodegeneration warrants further investigation.

There are several caveats that should be kept in mind when interpreting the results of this study. Although validation of *APP* in two independent cohorts of patients is a major advance in our study, unanticipated confounds may bias the results. For example, differences in blood counts and Parkinson's disease medications may bias gene expression results. Evaluation of *APP* expression in *de novo* Parkinson's disease patients and in a large well-characterized prospective study will be important to determine the validity of these results. Importantly, given that metabolic impairment plays an early role in the development of Parkinson's disease [38], determining whether *APP* expression is useful for distinguishing individuals at risk for Parkinson's disease, for progression of Parkinson's disease and/or for distinguishing sub-categories of Parkinson's disease patients will be important for future research.

Collectively, the findings provided in this study raises important biological questions. First, the knowledge of many disease comorbidities is limited and is primarily supported by epidemiological studies. In this regard, a potential link between Parkinson's disease and type 2 diabetes has been challenged by several epidemiological studies [39], [40] and the evidence of this association is not conclusive [41]. We overcome this challenge by demonstrating that Parkinson's disease and type 2 diabetes are highly interconnected at the molecular level. Importantly, given the involvement of *APP* in insulin regulation and neurodegeneration, its upregulation in blood of Parkinson's disease and type 2 diabetes provides a novel link between both diseases. Evaluation of *APP* as a potential predictor of neurodegeneration in type 2 diabetes is warranted. We foresee this study will provide a platform to generate novel hypothesis and therapeutic strategies for both devastating diseases. With the increasing amount of data deposited in disease databases, network biology provides a cost-effective tool for the discovery of biomarkers and therapeutic targets for validation.

## Supporting Information

Table S1 Genes identified by GWAS associated with Parkinson's disease. Genes with a genome-wide significance level of p<10^−08^ were included in this study.
(DOC)

Table S2 Genes identified in GWAS associated with type 2 diabetes. Genes with a genome-wide significance level of p<10^−08^ were included in this study.
(DOC)

Table S3 RWR scores for 200 top-ranked genes according to GPEC.
(DOC)

Table S4 Microarray data for the transcripts dysregulated in pre-diabetes, type 2 diabetes and Parkinson's disease. FC is the log 2-fold change.
(DOC)
