# Supplementary material for: Integrative Network Analysis Unveils Convergent Molecular Pathways in Parkinson's Disease and Diabetes
Source: PLoS One. 2013 Dec 20;8(12):e83940. doi: 10.1371/journal.pone.0083940 (PMC3869818; doi:10.1371/journal.pone.0083940)
Supplement: Table S1 — Genes identified by GWAS associated with Parkinson's disease. Genes with a genome-wide significance level of p<10−08 were included in this study. (DOC) [file pone.0083940.s001.doc]

Table S1. Genes identified by GWAS associated with Parkinson’s Disease.

| **Date added to catalog** | **PUBMED ID** | **Reported Genes** | **P-value** |
| --- | --- | --- | --- |
| 5/18/12 | 22438815 | *GAK, DGKQ* | 3E-12 |
| 5/18/12 | 22438815 | *MAPT, STH* | 8E-52 |
| 5/18/12 | 22438815 | *LRRK2* | 6E-15 |
| 5/18/12 | 22438815 | *SNCA* | 6E-65 |
| 5/18/12 | 22438815 | *SNCA* | 5E-11 |
| 5/18/12 | 22438815 | *LRRK2* | 3E-21 |
| 4/20/12 | 22451204 | *DGKQ* | 3E-09 |
| 4/20/12 | 22451204 | *SNCA* | 8E-35 |
| 4/20/12 | 22451204 | *LOC642072* | 3E-11 |
| 4/20/12 | 22451204 | *WNT3* | 3E-17 |
| 4/20/12 | 22451204 | *RIT2* | 2E-10 |
| 7/20/11 | 21738487 | *LRRK2* | 2E-28 |
| 7/20/11 | 21738487 | *GBA* | 5E-21 |
| 7/20/11 | 21738487 | *SNCA* | 2E-19 |
| 7/20/11 | 21738487 | *MAPT* | 3E-14 |
| 7/20/11 | 21738487 | *MCCC1, LAMP3* | 3E-10 |
| 7/20/11 | 21738487 | *SCARB2* | 8E-10 |
| 3/18/11 | 21292315 | *SYT11* | 4E-12 |
| 3/18/11 | 21292315 | *ACMSD* | 7E-09 |
| 3/18/11 | 21292315 | *STK39* | 4E-10 |
| 3/18/11 | 21292315 | *MCCC1, LAMP3* | 8E-12 |
| 3/18/11 | 21292315 | *GAK* | 4E-12 |
| 3/18/11 | 21292315 | *BST1* | 1E-16 |
| 3/18/11 | 21292315 | *SNCA* | 2E-47 |
| 3/18/11 | 21292315 | *HLA-DRB5* | 2E-14 |
| 3/18/11 | 21292315 | *LRRK2* | 6E-14 |
| 3/18/11 | 21292315 | *CCDC62, HIP1R* | 3E-13 |
| 3/18/11 | 21292315 | *MAPT* | 1E-28 |
| 1/3/11 | 21044948 | *SNCA* | 9E-16 |
| 1/3/11 | 21044948 | *MAPT* | 7E-12 |
| 9/22/10 | 20711177 | *HLA-DRA* | 2E-10 |
| 9/22/10 | 20711177 | *GAK* | 3E-09 |
| 9/22/10 | 20711177 | *SNCA* | 3E-11 |
| 12/10/09 | 19915576 | *PARK16, SLC45A3, NUCKS1, RAB7L1, SLC41A1, PM20D1* | 2E-12 |
| 12/10/09 | 19915576 | *BST1* | 3E-09 |
| 12/10/09 | 19915576 | *SNCA* | 7E-17 |
| 12/10/09 | 19915575 | *MAPT, C17orf69, KIAA1267, LOC644246* | 2E-16 |
| 12/10/09 | 19915575 | *SNCA* | 2E-16 |
| 12/10/09 | 19915575 | *NSF* | 1E-14 |
