# Supplementary material for: Integrative Network Analysis Unveils Convergent Molecular Pathways in Parkinson's Disease and Diabetes
Source: PLoS One. 2013 Dec 20;8(12):e83940. doi: 10.1371/journal.pone.0083940 (PMC3869818; doi:10.1371/journal.pone.0083940)
Supplement: Table S2 — Genes identified in GWAS associated with type 2 diabetes. Genes with a genome-wide significance level of p<10−08 were included in this study. (DOC) [file pone.0083940.s002.doc]

Table S2. Genes identified in GWAS associated with type 2 diabetes.

| **Date added to catalog** | **PUBMED ID** | **Reported Genes** | **P-value** |
| --- | --- | --- | --- |
| 6/26/13 | 23532257 | *ARF5, PAX4, SND1* | 2E-10 |
| 3/27/13 | 23300278 | *TCF7L2* | 3E-19 |
| 3/27/13 | 23300278 | *TCF7L2* | 6E-22 |
| 3/27/13 | 23300278 | *TCF7L2* | 3E-35 |
| 3/27/13 | 23300278 | *IGF2BP2* | 4E-09 |
| 3/27/13 | 23300278 | *TCF7L2* | 2E-38 |
| 3/27/13 | 23300278 | *IGF2BP2* | 2E-13 |
| 3/27/13 | 23300278 | *TCF7L2* | 9E-75 |
| 3/27/13 | 23300278 | *IGF2BP2* | 2E-19 |
| 3/19/13 | 23209189 | *TCF7L2* | 1E-35 |
| 11/15/12 | 22961080 | *GRK5* | 7E-09 |
| 11/15/12 | 22961080 | *RASGRP1* | 4E-09 |
| 11/15/12 | 22961080 | *GLIS3* | 6E-12 |
| 11/15/12 | 22961080 | *CDKN2B* | 3E-17 |
| 11/15/12 | 22961080 | *CDC123* | 7E-09 |
| 11/15/12 | 22961080 | *HNF1B* | 2E-11 |
| 11/15/12 | 22961080 | *FAM58A* | 2E-09 |
| 11/15/12 | 22961080 | *DUSP9* | 7E-16 |
| 11/15/12 | 22961080 | *CDKAL1* | 7E-10 |
| 8/7/12 | 22693455 | *LAMA1* | 8E-09 |
| 8/7/12 | 22693455 | *TCF7L2* | 2E-40 |
| 8/7/12 | 22693455 | *CDKAL1* | 7E-10 |
| 8/7/12 | 22693455 | *TCF7L2* | 4E-21 |
| 8/7/12 | 22693455 | *FTO* | 1E-20 |
| 8/7/12 | 22693455 | *CDKAL1* | 6E-11 |
| 8/7/12 | 22693455 | *HHEX* | 2E-09 |
| 8/7/12 | 22693455 | *IG2BP2* | 3E-09 |
| 2/7/12 | 22238593 | *RBM43, RND3* | 7E-09 |
| 1/18/12 | 22158537 | *MAEA* | 2E-20 |
| 1/18/12 | 22158537 | *GLIS3* | 2E-14 |
| 1/18/12 | 22158537 | *FITM2,R3HDML,HNF4A* | 1E-11 |
| 1/18/12 | 22158537 | *GCC1,PAX4* | 5E-11 |
| 1/18/12 | 22158537 | *PSMD6* | 8E-11 |
| 1/18/12 | 22158537 | *ZFAND3* | 2E-10 |
| 12/17/11 | 22101970 | *TCF7L2* | 2E-15 |
| 9/28/11 | 21874001 | *HMG20A* | 7E-11 |
| 9/28/11 | 21874001 | *AP3S2* | 2E-11 |
| 9/28/11 | 21874001 | *HNF4A* | 3E-10 |
| 8/18/11 | 21799836 | *KCNQ1* | 2E-17 |
| 6/3/11 | 21573907 | *CDKN2A, CDKN2B* | 6E-10 |
| 10/12/10 | 20862305 | *SPRY2* | 6E-09 |
| 9/28/10 | 20818381 | *C2CD4A,C2CD4B* | 9E-14 |
| 7/12/10 | 20581827 | *BCL11A* | 3E-15 |
| 7/12/10 | 20581827 | *ZBED3* | 3E-12 |
| 7/12/10 | 20581827 | *KLF14* | 2E-10 |
| 7/12/10 | 20581827 | *TP53INP1* | 1E-09 |
| 7/12/10 | 20581827 | *KCNQ1* | 3E-13 |
| 7/12/10 | 20581827 | *CENTD2* | 1E-22 |
| 7/12/10 | 20581827 | *HMGA2* | 4E-09 |
| 7/12/10 | 20581827 | *ZFAND6* | 2E-09 |
| 7/12/10 | 20581827 | *PRC1* | 2E-10 |
| 7/12/10 | 20581827 | *DUSP9* | 3E-10 |
| 7/12/10 | 20581827 | *IRS1* | 5E-20 |
| 7/12/10 | 20581827 | *MTNR1B* | 8E-15 |
| 7/12/10 | 20581827 | *IGF2BP2* | 2E-09 |
| 7/12/10 | 20581827 | *CDKAL1* | 2E-22 |
| 7/12/10 | 20581827 | *JAZF1* | 3E-09 |
| 7/12/10 | 20581827 | *CDKN2A,CDKN2B* | 1E-10 |
| 7/12/10 | 20581827 | *HHEX,IDE* | 1E-15 |
| 7/12/10 | 20581827 | *TCF7L2* | 2E-51 |
| 3/17/10 | 20174558 | *SRR* | 3E-09 |
| 3/17/10 | 20174558 | *PTPRD* | 9E-10 |
| 3/17/10 | 20174558 | *KCNQ1* | 1E-09 |
| 5/7/09 | 19401414 | *CDKAL1* | 7E-20 |
| 5/7/09 | 19401414 | *CDKN2A, CDKN2B* | 2E-29 |
| 5/7/09 | 19401414 | *KCNQ1* | 1E-26 |
| 5/7/09 | 19401414 | *SLC30A8* | 2E-14 |
| 5/7/09 | 19401414 | *HHEX* | 7E-12 |
| 5/7/09 | 19401414 | *TCF7L2* | 8E-12 |
| 1/15/09 | 19056611 | *FTO* | 2E-17 |
| 1/15/09 | 19056611 | *KCNJ11* | 1E-09 |
| 1/15/09 | 19056611 | *TCF7L2* | 6E-16 |
| 1/15/09 | 19056611 | *TCF7L2* | 9E-30 |
| 4/10/09 | 18711366 | *CDKAL1* | 3E-10 |
| 4/10/09 | 18711366 | *IGF2BP2* | 1E-09 |
| 4/10/09 | 18711366 | *KCNQ1* | 1E-16 |
| 4/3/09 | 18711367 | *KCNQ1* | 2E-42 |
| 11/25/08 | 18372903 | *JAZF1* | 5E-14 |
| 11/25/08 | 18372903 | *CDC123,CAMK1D* | 1E-10 |
| 11/25/08 | 18372903 | *TSPAN8,LGR5* | 1E-09 |
| 11/25/08 | 18372903 | *THADA* | 1E-09 |
| 11/25/08 | 18372903 | *CDKAL1* | 1E-11 |
| 11/25/08 | 18372903 | *TCF7L2* | 3E-23 |
| 11/25/08 | 17554300 | *TCF7L2* | 5E-12 |
| 11/25/08 | 17463246 | *CDKAL1* | 4E-11 |
| 11/25/08 | 17463246 | *HHEX* | 6E-10 |
| 11/25/08 | 17463246 | *IGF2BP2* | 2E-09 |
| 11/25/08 | 17463246 | *TCF7L2* | 2E-31 |
| 11/25/08 | 17463248 | *IGF2BP2* | 9E-16 |
| 11/25/08 | 17463248 | *CDKAL1* | 4E-11 |
| 11/25/08 | 17463248 | *CDKN2A,CDKN2B* | 8E-15 |
| 11/25/08 | 17463248 | *FTO* | 1E-12 |
| 11/25/08 | 17463248 | *HHEX* | 6E-10 |
| 11/25/08 | 17463248 | *KCNJ11* | 7E-11 |
| 11/25/08 | 17460697 | *CDKAL1* | 8E-09 |
| 11/25/08 | 17460697 | *TCF7L2* | 2E-10 |
| 11/25/08 | 17463249 | *KCNJ11* | 5E-11 |
| 11/25/08 | 17463249 | *FTO* | 7E-14 |
| 11/25/08 | 17463249 | *IGF2BP2* | 9E-16 |
| 11/25/08 | 17463249 | *TCF7L2* | 1E-48 |
| 11/25/08 | 17293876 | *TCF7L2* | 2E-34 |
