# Supplementary material for: Integrative Network Analysis Unveils Convergent Molecular Pathways in Parkinson's Disease and Diabetes
Source: PLoS One. 2013 Dec 20;8(12):e83940. doi: 10.1371/journal.pone.0083940 (PMC3869818; doi:10.1371/journal.pone.0083940)
Supplement: Table S3 — RWR scores for 200 top-ranked genes according to GPEC. (DOC) [file pone.0083940.s003.doc]

Table S3. RWR scores for 200 top-ranked genes.

| **Rank** | **Entrez ID** | **Gene Symbol** | **Score** |
| --- | --- | --- | --- |
| 1 | 967 | *CD63* | 4.45E-03 |
| 2 | 983 | *CDK1* | 4.26E-03 |
| 3 | 83878 | *USHBP1* | 2.18E-03 |
| 4 | 5894 | *RAF1* | 1.43E-03 |
| 5 | 5585 | *PKN1* | 1.21E-03 |
| 6 | 5594 | *MAPK1* | 9.54E-04 |
| 7 | 387 | *RHOA* | 8.98E-04 |
| 8 | 1387 | *CREBBP* | 8.71E-04 |
| 9 | 1315 | *COPB1* | 7.83E-04 |
| 10 | 207 | *AKT1* | 7.68E-04 |
| 11 | 375 | *ARF1* | 7.68E-04 |
| 12 | 673 | *BRAF* | 7.59E-04 |
| 13 | 5900 | *RALGDS* | 7.01E-04 |
| 14 | 377 | *ARF3* | 6.99E-04 |
| 15 | 351 | *APP* | 6.97E-04 |
| 16 | 5457 | *POU4F1* | 6.90E-04 |
| 17 | 9475 | *ROCK2* | 6.76E-04 |
| 18 | 5595 | *MAPK3* | 6.75E-04 |
| 19 | 5578 | *PRKCA* | 6.54E-04 |
| 20 | 6093 | *ROCK1* | 6.48E-04 |
| 21 | 5105 | *PCK1* | 6.18E-04 |
| 22 | 10993 | *SDS* | 6.02E-04 |
| 23 | 1019 | *CDK4* | 5.90E-04 |
| 24 | 6654 | *SOS1* | 5.87E-04 |
| 25 | 7410 | *VAV2* | 5.24E-04 |
| 26 | 5925 | *RB1* | 5.15E-04 |
| 27 | 5111 | *PCNA* | 5.07E-04 |
| 28 | 139189 | *DGKK* | 5.06E-04 |
| 29 | 1432 | *MAPK14* | 4.99E-04 |
| 30 | 5970 | *RELA* | 4.85E-04 |
| 31 | 8844 | *KSR1* | 4.77E-04 |
| 32 | 3074 | *HEXB* | 4.54E-04 |
| 33 | 1499 | *CTNNB1* | 4.52E-04 |
| 34 | 3073 | *HEXA* | 4.44E-04 |
| 35 | 1213 | *CLTC* | 4.37E-04 |
| 36 | 3551 | *IKBKB* | 4.37E-04 |
| 37 | 1956 | *EGFR* | 4.31E-04 |
| 38 | 324 | *APC* | 4.30E-04 |
| 39 | 5921 | *RASA1* | 4.25E-04 |
| 40 | 7369 | *UMOD* | 4.16E-04 |
| 41 | 1607 | *DGKB* | 4.11E-04 |
| 42 | 9610 | *RIN1* | 3.92E-04 |
| 43 | 701 | *BUB1B* | 3.83E-04 |
| 44 | 6850 | *SYK* | 3.62E-04 |
| 45 | 6300 | *MAPK12* | 3.59E-04 |
| 46 | 5701 | *PSMC2* | 3.29E-04 |
| 47 | 5702 | *PSMC3* | 3.28E-04 |
| 48 | 335 | *APOA1* | 3.28E-04 |
| 49 | 338 | *APOB* | 3.27E-04 |
| 50 | 7189 | *TRAF6* | 3.24E-04 |
| 51 | 10451 | *VAV3* | 3.20E-04 |
| 52 | 5441 | *POLR2L* | 3.19E-04 |
| 53 | 5588 | *PRKCQ* | 3.18E-04 |
| 54 | 2253 | *FGF8* | 3.17E-04 |
| 55 | 2932 | *GSK3B* | 3.13E-04 |
| 56 | 6714 | *SRC* | 3.10E-04 |
| 57 | 2099 | *ESR1* | 3.00E-04 |
| 58 | 23683 | *PRKD3* | 2.97E-04 |
| 59 | 7409 | *VAV1* | 2.90E-04 |
| 60 | 84033 | *OBSCN* | 2.86E-04 |
| 61 | 7124 | *TNF* | 2.77E-04 |
| 62 | 699 | *BUB1* | 2.77E-04 |
| 63 | 5581 | *PRKCE* | 2.73E-04 |
| 64 | 9578 | *CDC42BPB* | 2.71E-04 |
| 65 | 6790 | *AURKA* | 2.69E-04 |
| 66 | 367 | *AR* | 2.68E-04 |
| 67 | 1173 | *AP2M1* | 2.67E-04 |
| 68 | 28996 | *HIPK2* | 2.63E-04 |
| 69 | 5170 | *PDPK1* | 2.60E-04 |
| 70 | 8218 | *CLTCL1* | 2.60E-04 |
| 71 | 1211 | *CLTA* | 2.56E-04 |
| 72 | 65125 | *WNK1* | 2.54E-04 |
| 73 | 5584 | *PRKCI* | 2.47E-04 |
| 74 | 51701 | *NLK* | 2.46E-04 |
| 75 | 8907 | *AP1M1* | 2.45E-04 |
| 76 | 1021 | *CDK6* | 2.42E-04 |
| 77 | 7040 | *TGFB1* | 2.40E-04 |
| 78 | 5796 | *PTPRK* | 2.35E-04 |
| 79 | 10000 | *AKT3* | 2.31E-04 |
| 80 | 140803 | *TRPM6* | 2.30E-04 |
| 81 | 10053 | *AP1M2* | 2.26E-04 |
| 82 | 2931 | *GSK3A* | 2.26E-04 |
| 83 | 26750 | *RPS6KC1* | 2.25E-04 |
| 84 | 348 | *APOE* | 2.24E-04 |
| 85 | 6416 | *MAP2K4* | 2.20E-04 |
| 86 | 51384 | *WNT16* | 2.20E-04 |
| 87 | 5663 | *PSEN1* | 2.20E-04 |
| 88 | 3611 | *ILK* | 2.18E-04 |
| 89 | 23435 | *TARDBP* | 2.17E-04 |
| 90 | 5598 | *MAPK7* | 2.17E-04 |
| 91 | 2534 | *FYN* | 2.14E-04 |
| 92 | 89780 | *WNT3A* | 2.13E-04 |
| 93 | 1020 | *CDK5* | 2.12E-04 |
| 94 | 1436 | *CSF1R* | 2.10E-04 |
| 95 | 7204 | *TRIO* | 2.10E-04 |
| 96 | 80781 | *COL18A1* | 2.09E-04 |
| 97 | 1024 | *CDK8* | 2.09E-04 |
| 98 | 11021 | *RAB35* | 2.08E-04 |
| 99 | 157 | *ADRBK2* | 2.07E-04 |
| 100 | 1454 | *CSNK1E* | 2.07E-04 |
| 101 | 2200 | *FBN1* | 2.06E-04 |
| 102 | 23178 | *PASK* | 2.05E-04 |
| 103 | 55872 | *PBK* | 2.04E-04 |
| 104 | 1018 | *CDK3* | 2.03E-04 |
| 105 | 5604 | *MAP2K1* | 2.02E-04 |
| 106 | 29110 | *TBK1* | 2.02E-04 |
| 107 | 10595 | *ERN2* | 2.01E-04 |
| 108 | 8317 | *CDC7* | 1.99E-04 |
| 109 | 4139 | *MARK1* | 1.99E-04 |
| 110 | 9833 | *MELK* | 1.98E-04 |
| 111 | 51755 | *CDK12* | 1.97E-04 |
| 112 | 4811 | *NID1* | 1.97E-04 |
| 113 | 83732 | *RIOK1* | 1.97E-04 |
| 114 | 3482 | *IGF2R* | 1.96E-04 |
| 115 | 57538 | *ALPK3* | 1.95E-04 |
| 116 | 93664 | *CADPS2* | 1.95E-04 |
| 117 | 55561 | *CDC42BPG* | 1.95E-04 |
| 118 | 84254 | *CAMKK1* | 1.94E-04 |
| 119 | 7443 | *VRK1* | 1.94E-04 |
| 120 | 5580 | *PRKCD* | 1.93E-04 |
| 121 | 5579 | *PRKCB* | 1.89E-04 |
| 122 | 1147 | *CHUK* | 1.89E-04 |
| 123 | 4342 | *MOS* | 1.88E-04 |
| 124 | 127933 | *UHMK1* | 1.88E-04 |
| 125 | 1456 | *CSNK1G3* | 1.87E-04 |
| 126 | 57172 | *CAMK1G* | 1.87E-04 |
| 127 | 8899 | *PRPF4B* | 1.87E-04 |
| 128 | 5608 | *MAP2K6* | 1.86E-04 |
| 129 | 6011 | *GRK1* | 1.85E-04 |
| 130 | 25778 | *DSTYK* | 1.85E-04 |
| 131 | 10733 | *PLK4* | 1.85E-04 |
| 132 | 54101 | *RIPK4* | 1.85E-04 |
| 133 | 9641 | *IKBKE* | 1.85E-04 |
| 134 | 3654 | *IRAK1* | 1.85E-04 |
| 135 | 5587 | *PRKD1* | 1.84E-04 |
| 136 | 4214 | *MAP3K1* | 1.84E-04 |
| 137 | 1017 | *CDK2* | 1.84E-04 |
| 138 | 984 | *CDK11B* | 1.83E-04 |
| 139 | 176 | *ACAN* | 1.83E-04 |
| 140 | 255239 | *ANKK1* | 1.83E-04 |
| 141 | 817 | *CAMK2D* | 1.83E-04 |
| 142 | 146057 | *TTBK2* | 1.82E-04 |
| 143 | 8550 | *MAPKAPK5* | 1.82E-04 |
| 144 | 156 | *ADRBK1* | 1.82E-04 |
| 145 | 122011 | *CSNK1A1L* | 1.81E-04 |
| 146 | 84930 | *MASTL* | 1.81E-04 |
| 147 | 7867 | *MAPKAPK3* | 1.81E-04 |
| 148 | 204851 | *HIPK1* | 1.81E-04 |
| 149 | 65268 | *WNK2* | 1.81E-04 |
| 150 | 83931 | *STK40* | 1.81E-04 |
| 151 | 6199 | *RPS6KB2* | 1.81E-04 |
| 152 | 6249 | *CLIP1* | 1.80E-04 |
| 153 | 5292 | *PIM1* | 1.80E-04 |
| 154 | 5606 | *MAP2K3* | 1.79E-04 |
| 155 | 54986 | *ULK4* | 1.79E-04 |
| 156 | 8569 | *MKNK1* | 1.79E-04 |
| 157 | 9024 | *BRSK2* | 1.79E-04 |
| 158 | 5592 | *PRKG1* | 1.79E-04 |
| 159 | 29941 | *PKN3* | 1.79E-04 |
| 160 | 10298 | *PAK4* | 1.78E-04 |
| 161 | 8621 | *CDK13* | 1.78E-04 |
| 162 | 94 | *ACVRL1* | 1.78E-04 |
| 163 | 5261 | *PHKG2* | 1.78E-04 |
| 164 | 11011 | *TLK2* | 1.77E-04 |
| 165 | 494551 | *WEE2* | 1.77E-04 |
| 166 | 116988 | *AGAP3* | 1.77E-04 |
| 167 | 836 | *CASP3* | 1.77E-04 |
| 168 | 6732 | *SRPK1* | 1.77E-04 |
| 169 | 4117 | *MAK* | 1.77E-04 |
| 170 | 1311 | *COMP* | 1.77E-04 |
| 171 | 7059 | *THBS3* | 1.76E-04 |
| 172 | 5260 | *PHKG1* | 1.76E-04 |
| 173 | 139728 | *PNCK* | 1.76E-04 |
| 174 | 131890 | *GRK7* | 1.76E-04 |
| 175 | 5218 | *CDK14* | 1.76E-04 |
| 176 | 116987 | *AGAP1* | 1.76E-04 |
| 177 | 56924 | *PAK6* | 1.76E-04 |
| 178 | 112858 | *TP53RK* | 1.75E-04 |
| 179 | 22853 | *LMTK2* | 1.75E-04 |
| 180 | 23235 | *SIK2* | 1.75E-04 |
| 181 | 369 | *ARAF* | 1.74E-04 |
| 182 | 8997 | *KALRN* | 1.73E-04 |
| 183 | 5590 | *PRKCZ* | 1.72E-04 |
| 184 | 9262 | *STK17B* | 1.72E-04 |
| 185 | 9020 | *MAP3K14* | 1.72E-04 |
| 186 | 57551 | *TAOK1* | 1.71E-04 |
| 187 | 5129 | *CDK18* | 1.71E-04 |
| 188 | 8428 | *STK24* | 1.71E-04 |
| 189 | 8573 | *CASK* | 1.71E-04 |
| 190 | 2475 | *MTOR* | 1.71E-04 |
| 191 | 30811 | *HUNK* | 1.70E-04 |
| 192 | 8999 | *CDKL2* | 1.70E-04 |
| 193 | 65975 | *STK33* | 1.70E-04 |
| 194 | 85481 | *PSKH2* | 1.70E-04 |
| 195 | 340156 | *MYLK4* | 1.70E-04 |
| 196 | 646643 | *SBK2* | 1.70E-04 |
| 197 | 79934 | *ADCK4* | 1.70E-04 |
| 198 | 1198 | *CLK3* | 1.67E-04 |
| 199 | 4296 | *MAP3K11* | 1.66E-04 |
| 200 | 5582 | *PRKCG* | 1.65E-04 |
