# Supplementary material for: Integrative Network Analysis Unveils Convergent Molecular Pathways in Parkinson's Disease and Diabetes
Source: PLoS One. 2013 Dec 20;8(12):e83940. doi: 10.1371/journal.pone.0083940 (PMC3869818; doi:10.1371/journal.pone.0083940)
Supplement: Table S4 — Microarray data for the transcripts dysregulated in pre-diabetes, type 2 diabetes and Parkinson's disease. FC is the log 2-fold change. (DOC) [file pone.0083940.s004.doc]

Table S4. Microarray data for the transcripts dysregulated in pre-diabetes, type 2 diabetes and Parkinson’s disease. FC is the log 2 fold change.

| Transcript | Pre-diabetes  (log FC, p-value) | Type 2 diabetes  (log FC, p-value) | Parkinson’s disease  (log FC, p-value) |
| --- | --- | --- | --- |
| app | 1.4, 3E-03 | 1.7, 8E-04 | 2.2, 3E-03 |
| srrm2 | -1.0, 3E-03 | -0.8, 7E-04 | 0.3, 4E-02 |
| chpt1 | 1.1, 8E-04 | 1.0, 4E-04 | -0.3, 1E-03 |
| epb41 | 1.7, 1E-03 | 2.0, 5E-05 | -1.0, 5E-02 |
| gpr97 | 1.4, 9E-04 | 1.6, 3E-04 | 0.2, 1E-03 |
| ppm1a | 2.3, 3E-03 | 2.3, 7E-04 | -0.3, 2E-02 |
| bcl2l1 | 2.2, 8E-04 | 2.3, 4E-05 | -0.9, 4E-02 |
